# Mobility and Its Psychological Correlates: Subjective Age and Mobility-Related Behavioral Flexibility

**DOI:** 10.1093/geroni/igaf122.1847

**Published:** 2025-12-31

**Authors:** Christina Roecke, Minxia Luo, Zhiyong Zhou, Robert Weibel

**Affiliations:** University of Zurich, Zürich, Switzerland; University of Zurich, Zürich, Switzerland; University of Zurich, Zürich, Switzerland; University of Zurich, Zürich, Switzerland

## Abstract

Mobility is a key determinant of healthy aging and has been shown to be associated with older adults’ health and well-being. However, the psychological antecedents of mobility have been understudied. This study examined daily mobility in relation to two psychological constructs that have been found to be associated with older adults’ everyday engagement, including daily subjective age and trait-like attitudes of mobility-related behavioral flexibility. We examined data from 101 older participants (aged 65-88 years) who carried a GPS sensor and completed smartphone experience sampling surveys over two weeks. Participants reported their momentary subjective age seven times per day and reported the farthest place they travelled during the day each evening. Moreover, daily distance travelled from home was extracted from the GPS data. At baseline, participants completed a questionnaire on their mobility-related behavioral flexibility. Results from multilevel modelling showed that neither self-reported nor GPS-derived daily mobility was associated with daily subjective age within persons on average. However, moderation analysis indicated that for participants reporting higher flexibility concerning environmental and personal challenges, on days when feeling younger than their actual age, they reported to have travelled further away from home. The results remained the same after controlling for age, sex, subjective health, and health conditions. Higher mobility-related flexibility in combination with feeling younger on a day are associated with participants’ greater reported out-of-home mobility on the same day.

